# Bio-encapsulation of allergen-derivatives for specific immunotherapy

**DOI:** 10.3389/fpls.2025.1652246

**Published:** 2025-09-02

**Authors:** Fabian Schubert, Elsa Arcalís, Maximilian Kyral, Barbara Jeitler, Marianne Raith, Ines Swoboda, Eva Stoger

**Affiliations:** ^1^ Institute of Plant Biotechnology and Cell Biology, Department of Biotechnology and Food Sciences, BOKU University, Vienna, Austria; ^2^ Research Center Molecular Biotechnoloy, The Molecular Biotechnology Section, University of Applied Sciences, Vienna, Austria

**Keywords:** molecular farming, bioencapsulation, recombinant pharmaceuticals, storage organelles, allergen, plant-based production

## Abstract

Allergen-specific oral immunotherapy is a disease-modifying treatment already established for respiratory allergies and tested for the treatment of several food allergies, with promising clinical and immunological outcomes. However, orally administered allergens must pass through the gastrointestinal tract, where they are exposed to proteolytic digestion. This study describes the design of multi-layered protein bodies (PBs) in *Nicotiana benthamiana* as a platform for allergen encapsulation, offering potential advantages for oral immunotherapy. By co-expression of three zein variants we generated multi-layered PBs with distinct core and shell structures containing derivatives of the major fish allergen parvalbumin. The specific layering and structural integrity of the PBs were confirmed by confocal microscopy. Correlative light and electron microscopy (CLEM), combined with immunolabelling, was then used to verify the exact position of the allergens in the different layers of the PBs. *In vitro* experiments simulating the gastrointestinal digestion process revealed a significantly increased, layer-specific resistance of PB-encapsulated allergens compared to soluble allergens. Additionally, the uptake of PBs by human intestinal epithelial cells was simulated using Caco-2 cells. Our work provides further insight into protein storage organelle formation and novel bioencapsulation strategies to produce customized delivery vehicles, whose compartments may offer increased protection against enzymatic degradation and support prolonged persistence upon oral administration.

## 1 Introduction

Allergies, including food, seasonal, and skin allergies, affect a significant portion of the global population, with approximately 20-30% of people worldwide reporting at least one allergic condition (Alska et al., 2025). One major factor contributing to the development of allergies is the deregulation of immune tolerance. This initiates an exaggerated immune response (Baloh et al., 2022) to harmless substances such as food proteins, animal hair or pollen leading to allergic diseases (Maeda et al., 2019). Symptoms of allergic diseases range from sneezing, itching and other mild symptoms to severe consequences such as anaphylactic reactions (Deschildre and Lejeune, 2018). Allergic diseases tend to gradually worsen over time and develop into chronic conditions that can significantly impair a patient´s quality of life. Additionally, they place a considerable demand on the healthcare system, highlighting the crucial necessity for more effective therapeutic solutions (Cosme-Blanco et al., 2020; Fendrick and Baldwin, 2001). Traditional methods of allergy treatment focus primarily on allergen avoidance and symptom management through pharmacological intervention (Iweala et al., 2018; Tenero et al., 2023). However, these approaches fail to address the underlying causes of allergies and thus may not offer long-term improvement. This is why allergen-specific immunotherapy has been developed and remains the only disease-modifying therapy option for allergic diseases to date (Alvaro-Lozano et al., 2020). However, the standard subcutaneous allergen immunotherapy often has significant drawbacks (Incorvaia et al., 2023). These include the risk of local and systemic allergic reactions and the time-consuming nature of the therapy, which requires regular visits to a medical office over an extended period (Özdemir et al., 2023). Therefore, there is an increasing need to explore alternative routes of administration for allergens to enhance the convenience and safety of immunotherapy. One promising approach is oral allergen-specific immunotherapy (Jones et al., 2014). As an alternative to subcutaneous immunotherapy, oral administration of gradually increasing doses of allergens has shown considerable efficacy for achieving desensitization and immune tolerance (Iweala et al., 2018; Kulis et al., 2018). Additionally, oral administration can trigger a cascade where the interaction with antigen-presenting cells in the gastrointestinal tract induces regulatory T cells, leading to immunosuppression (Vickery et al., 2011). This process, known as oral tolerance, is facilitated by cytokines, which suppress inflammatory responses and prevent allergic reactions (Fu et al., 2006). Over time, repeated exposure to initially low-dose and gradually increasing amounts of the allergen can shift the immune response from an allergic Th2 profile to a more tolerogenic state, reducing hypersensitivity (Trogen et al., 2022). Beyond the exploration of alternative routes for allergen uptake, another critical focus for enhancing safety is the modification of allergens to create hypoallergenic variants. These variants are specifically designed to retain immunological activity while minimizing the potential to induce allergic reactions due to a reduced IgE binding capacity (Reginald et al., 2024). An example of this is the hypoallergenic version of parvalbumin, the major fish allergen (Swoboda et al., 2007). Although hypoallergenic parvalbumin offers the advantage of reducing allergic reactions, it also presents challenges. These include a reduced efficacy during oral immunotherapy, likely due to decreased stability (Freidl et al., 2020). This emphasizes the need for strategies to preserve the structural and functional integrity of orally administered allergens, especially during the gastrointestinal digestion phase. Encapsulating active components within protective matrices - such as liposomes, polymers, or protein-based carriers - mimic natural biological systems. One example is the structural protection of proteins within food matrices, which can enhance the stability and bioavailability of native allergens (Onwulata, 2012). A clinically relevant example is Palforzia^®^, an FDA-approved oral immunotherapy for peanut allergy. It consists of precisely dosed, defatted peanut flour administered with semi-solid food to promote immune tolerance through gradual allergen exposure while minimizing systemic allergic reactions (Berglund et al., 2017). In addition, encapsulation allows for more precise allergy management by enhancing allergen stability and potentially supporting prolonged persistence and exposure during gastrointestinal passage (Guo et al., 2021; Shutava and Lvov, 2012). It has been demonstrated with biopolymers that tailoring the structure and composition of the encapsulation matrix *in vitro* can influence the release kinetics and target specific sites within the body for allergen delivery (Drosou et al., 2017; Estevinho and Rocha, 2018). Therefore, the use of protective and biocompatible polymers may offer a useful strategy to improve the stability, safety and effectiveness of different oral delivery systems.

Zein, a prolamin protein, is the main storage protein in maize. The hydrophobic nature of zein promotes the formation of stable, self-assembled structures, called protein bodies (PBs). PBs have been successfully explored as drug carriers through *in vitro* loading (Lai and Guo, 2011; Liu et al., 2005). Due to its unique functional properties zein has also great potential for the encapsulation of allergens (Drosou et al., 2017; Schwestka et al., 2020). Various studies showed that zeins are resistant to digestion, indicating that the encapsulated allergens may remain intact until they reach target sites in the gastrointestinal immune system (Li et al., 2022; Zou and Gu, 2013). This slow and sustained release of allergens could be especially advantageous for oral immunotherapy, as it may help minimize the risk of systemic allergic reactions and increase the likelihood of uptake by gut-associated lymphoid tissues, which play a key role in inducing immune tolerance (Brayden et al., 2005). During normal biological processes, endogenous zein PBs form intracellularly in maize endosperm tissues, but ectopic PBs can also be induced in vegetative organs such as leaves (Llop-Tous et al., 2010). Given that plants are excellent production systems for recombinant therapeutic proteins and have the natural ability to form PBs, it is appealing to utilize plant hosts for *in vivo* microencapsulation. This can be done by directly incorporating recombinant proteins into protein storage organelles (Hofbauer et al., 2016; Whitehead et al., 2014). We have recently shown that the formation of multi-layered ectopic PBs in *N. benthamiana* leaves can be induced by selected combinations of zeins (Schwestka et al., 2023). In particular, the N-terminal part of 27-kDa-γ-zein, also known as Zera, directs the fused protein into the outer shell of the PBs, while the fusion to the 15-kDa-β-zein targets it to the core (Schwestka et al., 2023). We speculate that the encapsulation of allergens within these specialized structures may add an innovative advantage to oral immunotherapy by increasing allergen stability and enabling prolonged persistence throughout the digestive tract.

In order to establish multi-layer zein PBs as an effective bio-encapsulation platform for allergens, it is important to clarify several key questions. These include whether allergens can be directed to distinct protein body layers (PB layers), how this affects their digestive stability during gastric and intestinal phases and whether multi-layered PBs are efficiently taken up by intestinal cells. In the present study, we employ *Nicotiana benthamiana* for in-planta bio-encapsulation of both wild-type and hypoallergenic (mutant) parvalbumin to enhance their persistence in the gastrointestinal tract. We demonstrate the incorporation of the allergens into two distinct PB layers and confirm their uptake into intestinal epithelial cells. Additionally, we evaluate the protective effect of layer-specific encapsulation through simulated digestion.

## 2 Materials and methods

### 2.1 Constructs for plant transformation

A synthetic sequence coding for carp parvalbumin Cyp c 1 wild-type (CW, accession number AJ292211), was ordered in a pUC57 vector backbone (GeneCust, Boynes, France). Mutations at amino acid positions 52 and 93, as well as 54 and 91 – previously identified as key substitutions for generating a hypoallergenic variant (Swoboda et al., 2007) – were introduced via site-directed mutagenesis using two rounds of PCR with mismatched primers. Primers 5´-AGG TCT CGT CTG CTG GAG ATG GCA AGA TTG GAG-3´ and 5´-AGG TCT CAC TTG GGC AAT GAC AGC AAA GGC CTT C-3´ were used to introduce mutations at codons 93 and 52, respectively. Similarly, primers 5´-AGG TCT CCC AAG CCA AGA GCG GCT TCA TTG AG-3´ and 5´-AGG TCT CAC AGA GGC TCC AGC TTT CAG GAA GGC-3´ were used to target codons 54 and 91.

The mutated sequences were digested with BsaI and ligated, forming a new plasmid containing the carp mutant (CM) sequence. Next, we utilized pre-existing zein-fluorophore fusion vectors containing the fusion proteins 15-kDa-β-zein-mCherry and the N-terminal part of 27-kDa-γ-zein also known as Zera-EGFP (Schwestka et al., 2023). These vectors were digested with Kpn2I and SalI to replace the fluorophore coding regions by the wild-type and mutated allergen sequences (Schwestka et al., 2023), resulting in the final constructs Zera-CWF (pTRA-Zera-CWF), Zera-CMF (pTRA-Zera-CMF), 15-kDa-β-zein-CWF (pTRA-bz15-CWF) and 15-kDa-β-zein-CMF (pTRA-bz15-CMF) (**Supplementary Figure S1**).

### 2.2 Biological material and transformation

**Figure 1:**
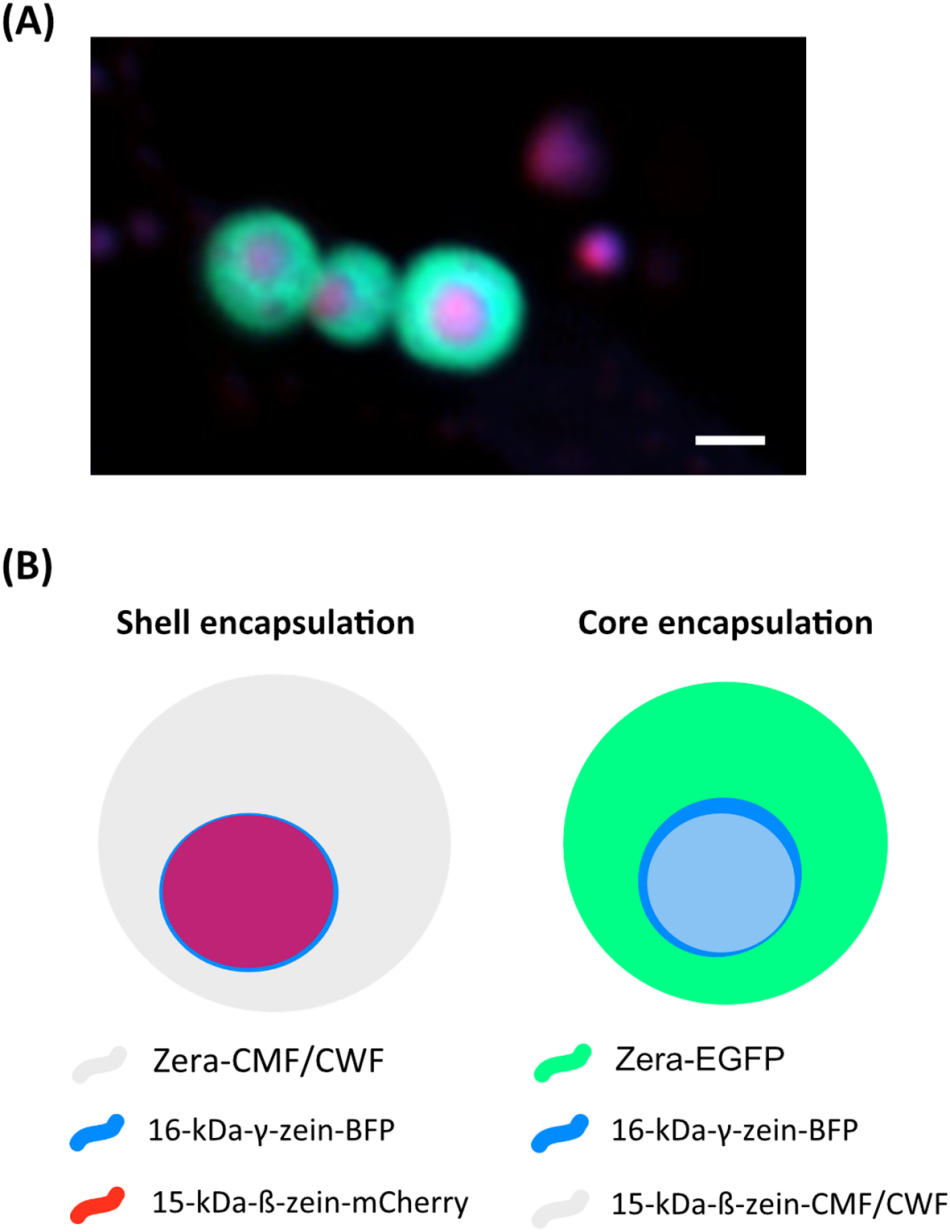
Encapsulation strategy of wild-type (CWF) and mutant parvalbumin fusions (CMF) in multilayered protein bodies. **(A)** Fluorescent protein bodies (Zera-EGFP, 16-kDa-γ-zein-BFP and 15-kDa-ß-zein-mCherry, as previously also described in Schwestka et al., 2023). The outer shell is indicated by GFP, while the core contains both BFP and mCherry. Notably, the BFP and mCherry signals do not fully co-localise. **(B)** Encapsulation strategies for CWF/CMF, derived from the setup shown in **(A)** Bar 1 µm.

*N. benthamiana* plants were grown in soil under a 16-hour photoperiod with 70% relative humidity and temperatures of 26°C during the day and 16°C at night. The plants were cultivated for a minimum of 4 weeks in this environment. Plant expression vectors were transferred into chemically competent *Rhizobium radiobacter* (commonly known and hereafter referred to as *Agrobacterium tumefaciens)* GV3101 containing the helper plasmid pMP90RK (Koncz and Schell, 1986). *A. tumefaciens* cultures containing the above-described constructs as well as cultures containing 15-kDa-β-zein-mCherry, 16-kDa-γ-zein-BFP and the N-terminal part of 27-kDa-γ-zein-EGFP (Zera-EGFP) (Schwestka et al., 2023), were inoculated from glycerol cryo-stocks and grown in Yeast Extract Beef Broth (YEB medium: 5 g/L beef extract, 1 g/L yeast extract, 5 g/L peptone, 5 g/L sucrose, 0.5 g/L MgSO4, sterilized by autoclaving) with 20 mg/L rifampicin and 50 mg/L carbenicillin at 28°C, shaking at 200 rpm. Subsequently, cultures were pelleted by centrifugation at 3000 x g for 10 minutes and washed twice with infiltration medium (10 mM MES pH 5.6, 10 mM MgCl_2_, 100 μM acetosyringone). The following *A.tumefaciens* strains were adjusted to an OD_600_ of 0.1 and combined prior to infiltration: Zera-CWF/CMF, 16-kDa-γ-zein-BFP and 15-kDa-β-zein-mCherry (*shell encapsulation*) and Zera-EGFP, 16-kDa-γ-zein-BFP and 15-kDa-β-zein-CMF/CWF (*core encapsulation*) (**Figure 1B**).

### 2.3 Extraction of protein bodies

Leaves were harvested 7 days post-infiltration (dpi) and homogenized into a fine powder with liquid nitrogen. The homogenate was suspended in phosphate buffered saline (PBS) supplemented with 2% Triton X-100, centrifuged for 20 minutes at 4300 rpm and the resulting pellet was subjected to several washes with PBS. The suspension was then passed through nylon membranes with decreasing pore sizes (180, 120, 60, 30 and 10 µm, Merck Millipore Ltd. Nylon, Burlington, USA) and the resulting filtrate was pelleted and resuspended in PBS. PBs were subsequently purified according to Schwestka et al., 2020 and Schwestka et al., 2023. Finally, the resuspended PBs were sonicated and loaded onto a CsCl cushion (density 1.45 g/cm3). The top layer containing the PBs was recovered, washed twice with PBS, and the final pellet was resuspended in PBS for subsequent use in the digestion and uptake assays.

### 2.4 Protein immunoblot analysis

Leaf tissue was harvested 7 dpi from plants transiently expressing either carp wild-type parvalbumin (CWF) or the mutant version (CMF), immediately frozen and ground to a fine powder. The samples were extracted with four volumes (w/v) of chilled extraction buffer (2% Triton X-100 in PBS) and incubated on ice for 15 minutes. The supernatant was used for immunoblotting and the pellet was resuspended in 1.5x reducing Laemmli buffer, incubated at 37°C for 1 hour and centrifuged at 16,000 x g for 5 minutes prior to immunoblot analysis. A polyclonal antiserum against the flag-tag was used as a primary antibody at a 1:20,000 dilution, followed by an anti-rabbit-HRP antibody at the same dilution (Promega, Madison, USA). Samples were analyzed in three independent biological replicates.

For suspensions of isolated PB as described in 2.3, samples were mixed with 4x reducing Laemmli buffer and heated at 80°C for 5–10 minutes. Immunoblot analysis was performed according to standard protocols and as described previously (Schwestka et al., 2023). Rabbit antiserum raised against the carp parvalbumin mutant – produced according to the protocol described by Freidl et al., 2020 and manufactured by Charles River Laboratories, Miserey, France - was diluted 1:20,000 and used as the primary antibody, recognizing both the wild-type and the mutant forms of the allergen (Freidl et al., 2020). An anti-rabbit-HRP antibody diluted 1:20,000 (Promega, Madison, USA) was used as a secondary antibody. The analyses were performed in three biological replicates.

### 2.5 Flow cytometry

PBs were isolated from 16.8 g leaf material and resuspended in one mL of PBS. The particles were quantified using a flow cytometer (CytoFlex S; Beckman Coulter, Brea, USA) in a V-bottom 96-well plate, with 50,000 events recorded per sample. GFP and mCherry fluorescence were excited at 488 nm and 561 nm, respectively, and emissions were detected at 525 nm (GFP) and 610 nm (mCherry). Gain settings were configured to 25 for forward scatter, 4 for side scatter, 8 for GFP, and 41 for mCherry. Each sample was analyzed at three dilutions (1:10, 1:100, and 1:1000), with measurements performed in triplicates.

### 2.6 Confocal laser scanning microscopy

Live cell imaging was used to assess the expression and deposition of labelled proteins into PBs as previously described (Schwestka et al., 2023). Thus, purified PB suspension or small pieces of infiltrated leaves (7 dpi) were examined under a Leica SP8 or a Zeiss LSM980 confocal microscope. Representative images from at least three biological replicates were analyzed using the softwares Leica LASX (Leica Microsystems, Wetzlar, Germany) or Zeiss ZEN lite (Carl Zeiss Microscopy, Jena, Germany).

#### 2.6.1 Localization of encapsulated CWF/CMF

In order to investigate the localization of the allergen fusions in the *shell encapsulation*, immunofluorescence analysis was performed on fresh tissue. Small tissue sections were excised from infiltrated leaves (7 dpi) with a razor blade and fixed in 4% paraformaldehyde in cacodylate buffer (0.1 M, pH 7.4) for 2h at room temperature. After several washes in cacodylate buffer (0.1 M, pH 7.4), cross sections (120 µm) were obtained with a vibratome. Subsequently, sections were dehydrated and rehydrated through an ethanol series, washed in phosphate buffer (0.1 M, pH 7.6) and incubated in 2% (w/v) cellulase Onozuka R-10 (from *Trichoderma viride*) in phosphate buffer (0.1 M, pH 7.4) for 1 h at room temperature. Subsequently, the sections were incubated in 5% (w/v) BSA (Fraction V) in phosphate buffer to block non-specific binding sites, followed by overnight incubation at 4°C with a 1:200 dilution of serum from an immunized rabbit that recognizes both the wild-type and the mutant forms of the allergen (Freidl et al., 2020). The immunostaining was visualized using an Alexa Fluor 488-conjugated donkey anti-rabbit antibody (Thermo Fisher Scientific, Waltham, USA). Sections were then mounted in 50% glycerol in PBS and observed in a confocal laser scanning microscope LSM980-AiryScan2 (Carl Zeiss Microscopy, Jena, Germany).

For the *core encapsulation* of the allergen, a visualization strategy distinct from the one used for the shell encapsulation was employed, allowing access to the core of the purified PBs. The PBs were high pressure frozen and freeze substituted as described in Huber et al., 2024. Briefly, 100 µL of PB suspension were pelleted by centrifugation, and subsequently dried at room temperature for 1 hour before the pelleted material was placed into the wells of aluminum Type B carriers (Science Services, Munich, Germany), with 1-hexadecene as a cryoprotectant. The samples were then high pressure frozen and freeze substituted in 0.2% glutaraldehyde and 0.2% uranyl acetate in anhydrous acetone. Samples were further infiltrated and embedded in HM20 resin (Polysciences, Warrington, USA) and then polymerized under UV light. Sections showing silver interferences were collected onto formvar coated finder grids to facilitate the correlation of the regions of interest for correlative light and electron microscopy (CLEM).

To enable precise localization of the encapsulated allergen within these resin-embedded PBs, sections were subjected to immunogold labelling, as described in Schwestka et al., 2023. In short, sections were blocked with 5% (w/v) bovine serum albumin in 0.1M phosphate buffer (pH 7.4) and incubated with the rabbit antiserum raised against the carp parvalbumin mutant (Freidl et al., 2020). Gold-conjugated donkey-anti-rabbit antibodies were used for visualization.

For CLEM, grids were first examined under the confocal microscope as described by Chambaud et al., 2023 and several regions of interest were selected. Following, grids were carefully recovered, air dried and the previously selected regions of interest were imaged under a FEI Tecnai G2 transmission electron microscope (TEM). Natural landmarks such as shape and size of the PBs were used to correlate confocal and TEM images using the landmark correlation tool of ImageJ and the ec-CLEM plugin (Paul-Gilloteaux et al., 2017) of the Icy software (De Chaumont et al., 2012). Representative images showing GFP fluorescence and immunogold from 3 biological replicates are presented.

### 2.7 PB uptake into Caco-2 cells

To study cellular uptake of PBs, Caco-2 cells (HTB-37, ATCC, Manassas, USA) were utilized. Cells were cultured according to the standard procedure described by Wanes et al., 2021 on ACLAR^®^ fluoropolymer foil (Science Service, Munich, Germany) pre-treated with UV light for 30 minutes to reduce potential contaminants. Approximately 70,000 PBs per cm², suspended in PBS and quantified by flow cytometry, were incubated with the cells for 24 hours. Following incubation, the medium was removed, and the cells were washed twice with PBS and subjected to confocal laser scanning microscopy (CLSM) or further prepared for electron microscopy. For confocal microscopy, cells were incubated in CellBrite^®^ for 45 minutes and then fixed in 2% paraformaldehyde (PFA). Samples were washed twice in PBS prior to imaging under a Leica SP8 CLSM. For electron microscopy, Caco-2 cells growing on ACLAR^®^ foil were fixed by cutting small pieces of foil (2x2 mm), immediately fixed in 2.5% glutaraldehyde and 2% paraformaldehyde in cacodylate buffer (0.15 M pH 7.4), and further processed as described in Arcalís et al., 2020. In short, double osmium impregnation was applied by post-fixing the cells in 2% osmium tetroxide added with 0.2% ruthenium red in 0.15 M cacodylate buffer, followed by thiocarbohydrazide solution (1% w/v in dH2O) and an additional incubation with 2% osmium tetroxide in dH2O. Subsequently, cells were incubated in UAR-EMS Uranyl Acetate Replacement Stain (1:4 v/v), followed by Walton’s lead aspartate stain (20 mM lead nitrate in 30 mM L-aspartic acid solution). Next, samples were dehydrated through an ethanol series with a final step in pure acetone. Samples were then progressively infiltrated in LV Resin, embedded and polymerized at 60°C for 48 h. Resulting blocks were trimmed and sectioned. 200 nm sections were mounted on silicon wafers and an SEM stub prior to imaging under an Apreo SEM (Thermo Fisher Scientific, Waltham, USA), operating in Optiplan mode (2kV, 0.1 nA).

### 2.8 Digestion stability assay

To test the digestive stability of the encapsulated proteins, we adapted the protocol from Moreno et al., 2005 by scaling down the reaction volume from 2.2 mL to 1.6 mL. The soluble wild-type (CW) and mutant (CM) allergens were adjusted to a concentration of 1.59 mg/mL. To determine the amount of leaf tissue required to obtain comparable starting quantities of PB-encapsulated allergen, immunoblot analyses were performed. The results indicated that 20 mg of leaf tissue contained approximately 5 µg of PB-encapsulated allergen. Before starting the digestive stability assay, aliquots from CWF and CMF were taken to establish a reference value at timepoint 0 (G-0). The digestion procedure was carried out under defined simulated gastric conditions (182 U Pepsin/mg protein in 1x simulated gastric fluid containing 0.15 M NaCl, pH 3) and simulated intestinal conditions (34.5 U trypsin/mg protein and 0.44 chymotrypsin/mg protein, 25 mM Bis-Tris and 9.2 mM CaCl2, pH 7). After stopping the digests, all aliquots were immediately frozen at -20°C until further analysis by immunoblotting. Detection was performed using a rabbit antiserum raised against mutant carp parvalbumin (Freidl et al., 2020) and a secondary anti-rabbit-HRP antibody. For the evaluation of the immunoblot results, band intensity profiles were analyzed using ImageLab software (Bio-Rad Laboratories, Inc, Hercules, USA). The signal intensity of each band was normalized to the reference band at G-0 and plotted against the total digestion time. Quantitative estimates were based on a minimum of three independent biological replicates (n = 3), and data were analyzed using GraphPad Prism (GraphPad Software, LLC, San Diego, USA). Statistical significance was assessed using two-way ANOVA followed by Dunnett´s multiple correction.

## 3 Results

### 3.1 Encapsulation of parvalbumin within multi-layered protein bodies

Based on previous studies by Schwestka et al., 2023, we employed a combination of 16-kDa-γ-zein, Zera (partial 27-kDa-γ-zein), and 15-kDa-β-zein to induce the *in vivo* formation of multi-layered protein bodies with distinct layers in *Nicotiana benthamiana*. To facilitate visual tracking, two of the zeins were fused to fluorescent proteins. The third (either 15-kDa-β-zein or Zera) was fused to carp parvalbumin variants, causing the incorporation of the fish allergen either in the core or the shell of the PBs (**Figure 1**).

**Figure 2:**
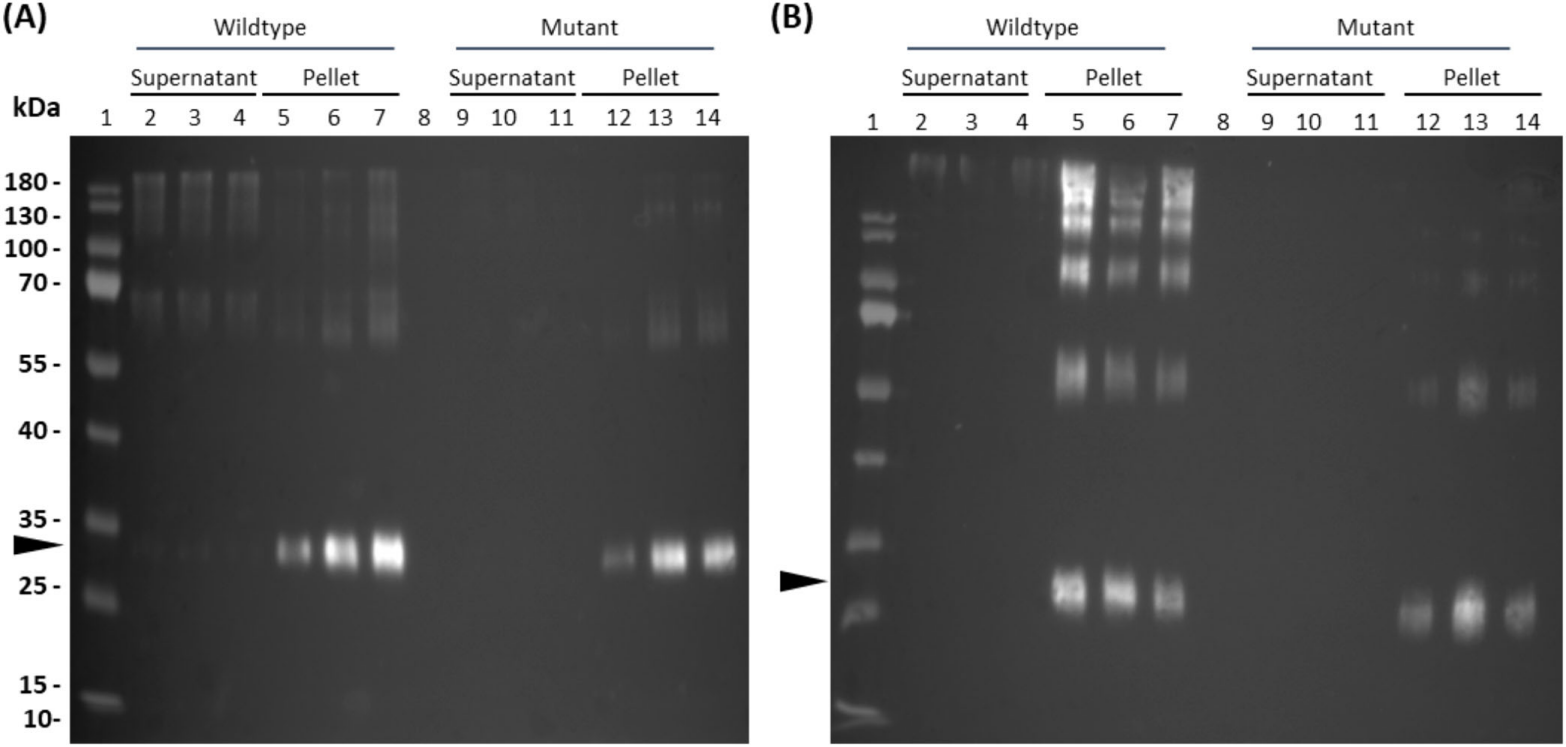
Detection of parvalbumin-derivatives from multilayered PBs of crude leaf extracts (7 dpi). Flag-tagged parvalbumin wild-type and mutant proteins were detected in immunoblots of crude leaf extracts using a polyclonal antibody against the flag-tag. **(A)** Core-encapsulation of the 15-kDa-β- zein-CWF (lanes 2-7) and 15-kDa-β- zein-CMF (lanes 9-14) fusion protein. **(B)** Shell-encapsulation of the Zera-CWF (2-7) and Zera-CMF (9-14) fusion protein. The arrows indicate the expected molecular mass of the fusion proteins 15-kDa-β- zein-CMF and 15-kDa-β- zein-CWF **(A)** and Zera-CWF and Zera-CMF **(B)**.

In order to encapsulate the allergen within the core of insoluble protein bodies, wild-type parvalbumin (CWF) or its hypoallergenic version (CMF) were fused to 15-kDa-β-zein. To achieve incorporation in the PB shell, they were fused to Zera (**Supplementary Figure S1**). We then co-expressed each of the zein-allergen fusion proteins with the two fluorescently labelled complementary zeins to obtain multi-layered PBs. Seven days after infiltration of *N. benthamiana*, the crude leaf extract was separated into soluble and pellet fractions as described in Schwestka et al., 2023 and analyzed by immunoblot detection (**Figure 2**). Distinct allergen-containing bands at 25 kDa (Zera-CWF/CMF) and 30 kDa (15-kDa-β-zein-CMF/CWF), along with some higher molecular mass bands likely representing incompletely reduced polymers, appeared only in the PB-containing pellet fractions (**Figure 2**). In the supernatants, only faint bands could be detected, confirming the incorporation of the wild-type and mutant parvalbumin into the PBs. To further confirm these findings, immunoblot analysis was also performed with the enriched PB fractions. These fractions displayed a banding pattern similar to that observed in the crude extract, with strong signals for both the Zera- and 15-kDa-β-zein fusion proteins at the respective positions (**Supplementary Figure S2**).

**Figure 3:**
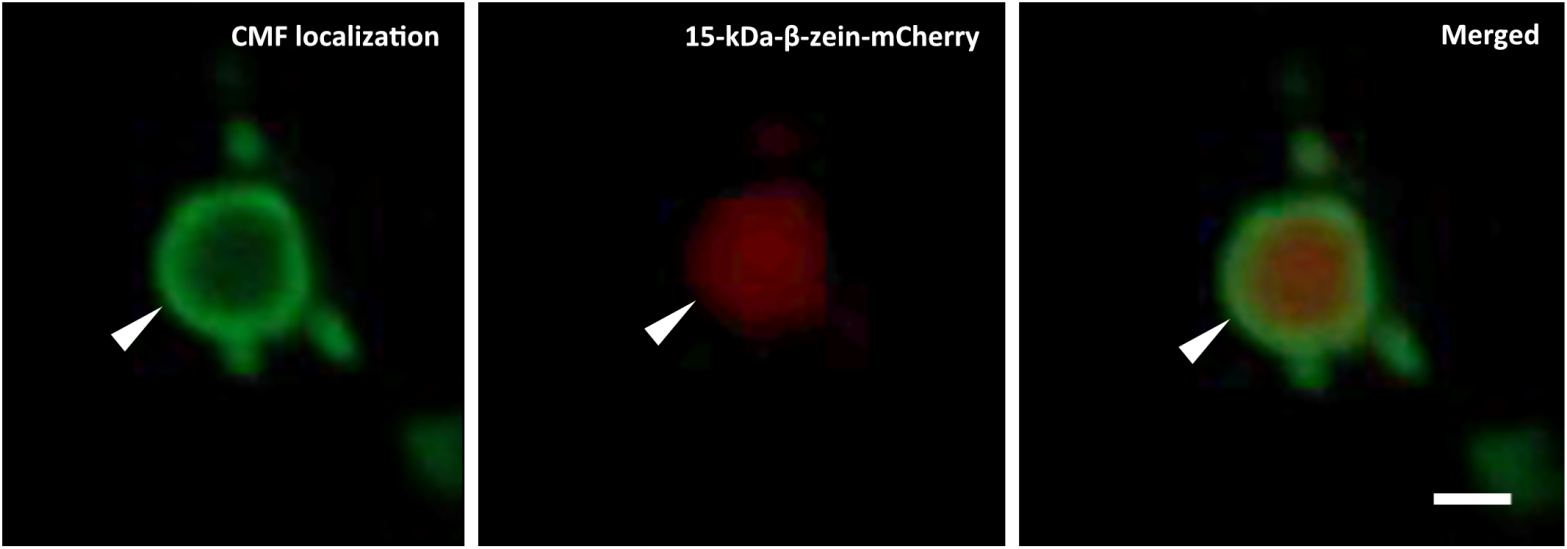
Shell encapsulation of mutant parvalbumin. Co-expression of Zera-CMF, 16-kDa-γ-zein-BFP, and 15-kDa-β-zein-mCherry. CMF localization was assessed by immunolocalization carried out on fixed vibratome sections. Rabbit serum recognizing both the wild-type and mutant form of the allergen served as primary antibody, followed by an Alexa Fluor 488-conjugated secondary antibody. First panel: Immunolocalization of CMF in the shell of the protein body completely surrounding the β-zein core; second panel: 15-kDa-β-zein-mCherry. Overlay images in the third panel reveal the spatial distribution of the signals within the PBs. CLSM images. Bar 1 µm.

**Figure 4:**
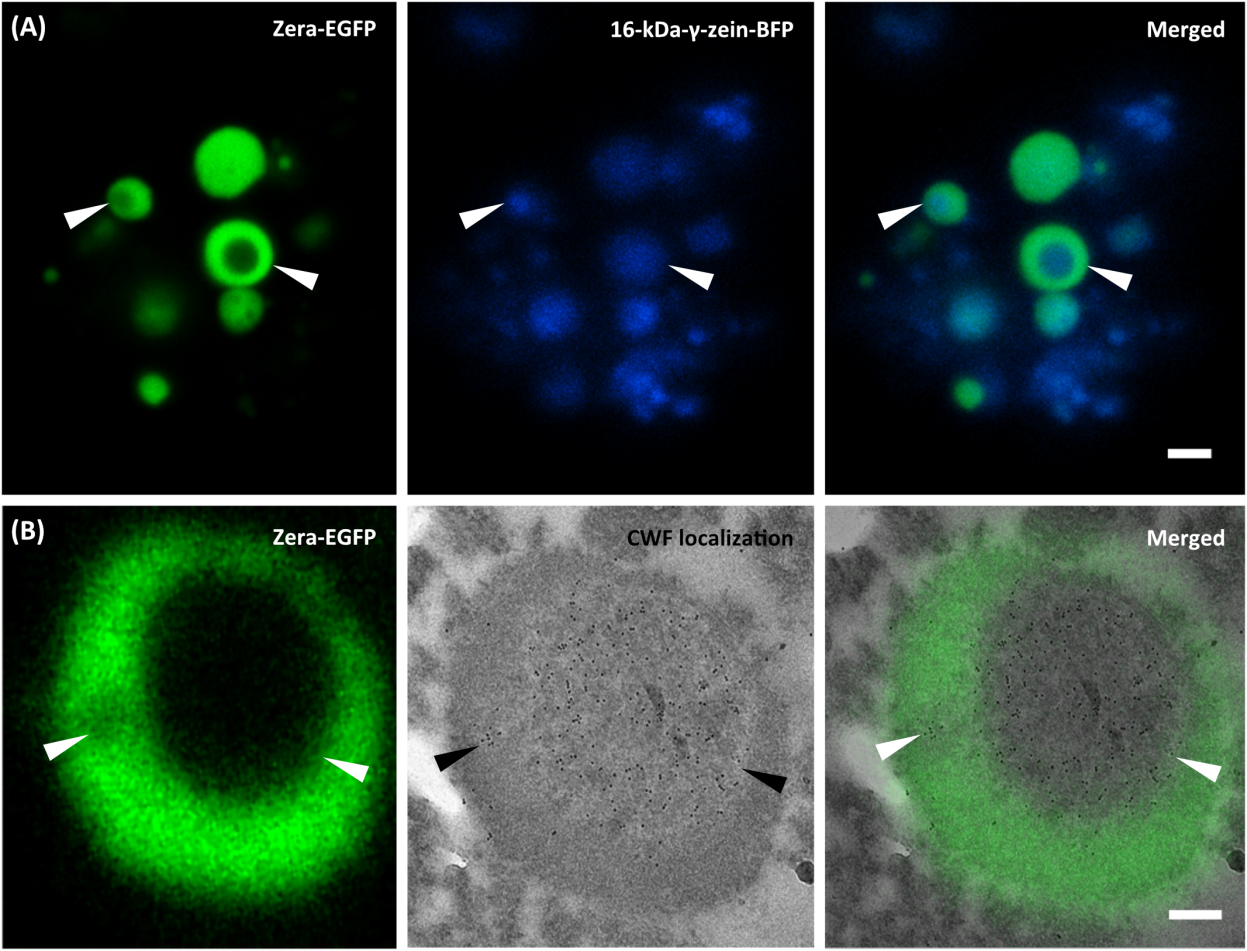
Core encapsulation of the wild-type parvalbumin. Co-expression of Zera-EGFP, 16-kDa-γ-zein-BFP, and 15-kDa-β-zein-CWF. **(A)** CLSM images of isolated PBs. First panel: Zera-EGFP; second panel: 16-kDa γ-zein-BFP; third panel: overlay of both channels. **(B)** First panel: CLSM image showing Zera-EGFP in resin-embedded PBs, prepared by high-pressure freezing and freeze substitution; second panel: CLEM image showing detection of wild-type parvalbumin via anti-parvalbumin antibody and 10 nm gold-labeled donkey anti-rabbit secondary antibodies. The third panel (overlay) reveals the spatial distribution of signals within the PBs. Bars 2 µm **(A)**, 0.25 µm **(B)**.

The incorporation of the allergen within PBs was further investigated by correlative light and electron microscopy, taking advantage of the fluorescent protein tags. For the shell encapsulation of the allergen, PBs in the cytoplasm of *N. benthamiana* revealed a red and blue fluorescent core (≤1 µm in size), representing the 15-kDa-β-zein-mCherry and 16-kDa-γ-zein-BFP (**Supplementary Figures S3**, **S4A**). The presence of parvalbumin was determined via immunofluorescence (**Figure 3**), which revealed a ring-shaped distribution of the allergen surrounding the fluorescent core. This is consistent with the expected localization of a Zera-fused protein within a multilayered PB measuring ≥1 µm in diameter (**Figure 1A**) (Schwestka et al., 2023). No significant differences in localization within the PB were observed between wild-type parvalbumin and its mutant variant (**Supplementary Figure S4B**). In the case of the core encapsulation, green fluorescent protein bodies (≥1 µm in size) were detected in the transformed leaf cells, showing a central void with a signal for 16-kDa-γ-zein-BFP (**Figure 4A**; **Supplementary Figure S4C**). For the localization of the allergen, we opted for immunogold labelling of thin sections of the enriched PB pellet followed by electron microscopy in order to gain access to the core of the PB. The preservation of GFP fluorescence after HPF-FS facilitated the identification of the protein bodies within the section. The central areas of the protein bodies were devoid of green fluorescence and showed abundant gold probes instead, indicating the presence of the allergen within the protein body core (**Figure 4B**). No co-localization with GFP was observed, confirming a precise distribution of the different components within the PB (Schwestka et al., 2023).

### 3.2 Simulated gastrointestinal digestion

**Figure 5:**
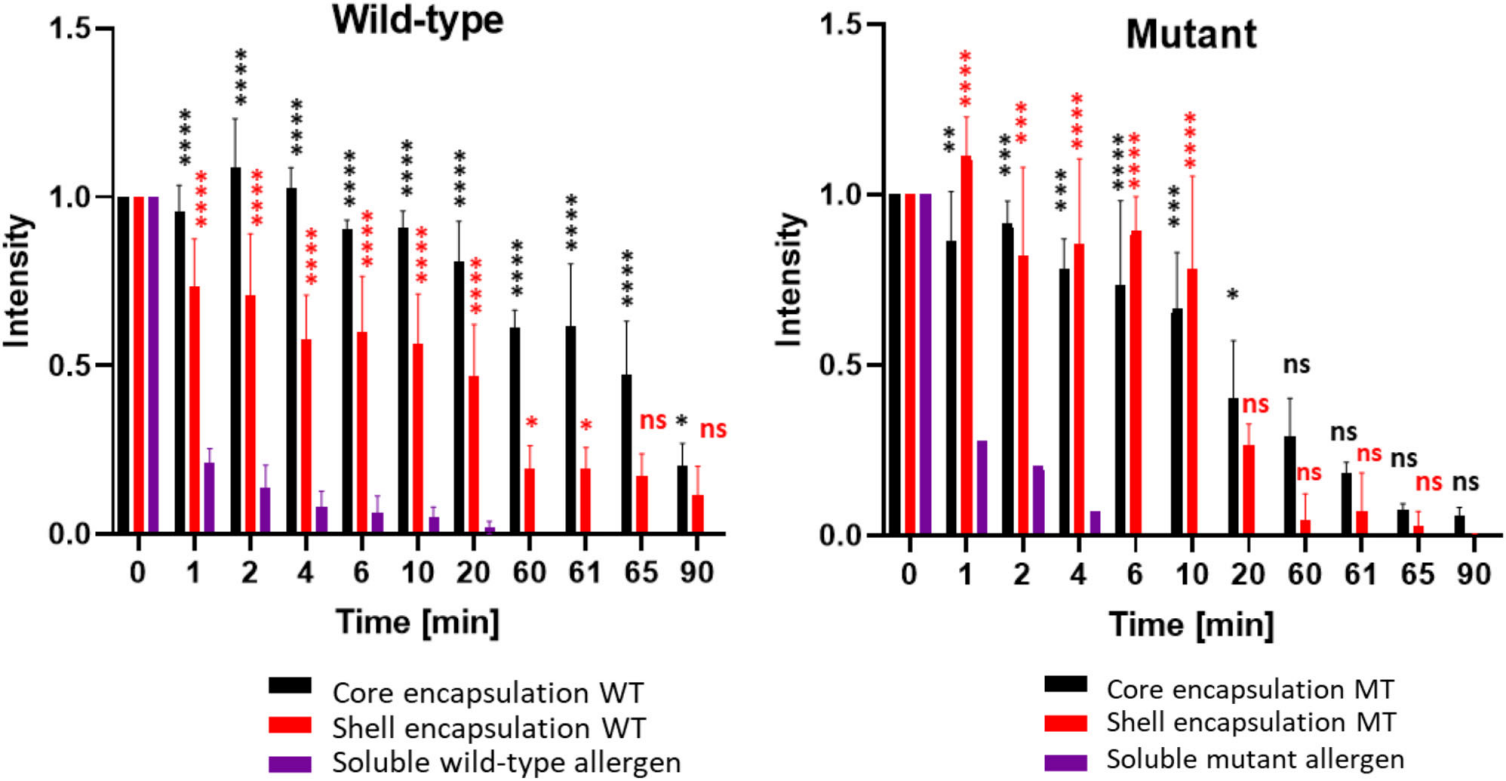
Encapsulated wild-type and mutant parvalbumins show higher resistance to simulated gastrointestinal digestion than the soluble proteins. The y-axis represents the relative signal strength compared to G-0 of the respective immunoblot. Detection was carried out using a rabbit antiserum recognizing both the mutant and wildtype carp parvalbumin. The x-axis represents the total digestion time in minutes. Gastric digestion was always started at G-0 by addition of pepsin to the samples (aliquots were taken after 1, 2, 4, 6, 10, 20, and 60 minutes). After 60 minutes, pepsin from the gastric phase was neutralized, and trypsin/chymotrypsin were added to the samples to initiate the intestinal phase (aliquots were taken after 61, 65 and 90 minutes). The values derived from three independent digests are shown (n = 3). A t-test to compare the allergen containing PBs with the soluble allergens was performed. Ns p > 0.05, *p ≤ 0.05, **p ≤ 0.01, ***p ≤ 0.001, ****p ≤ 0.00001.

To assess whether bio-encapsulation enhances allergen persistence under proteolytic conditions, we first used soluble wild-type (CW) and mutant parvalbumin (CM) as controls in a simulated digestion process. Following pepsin addition, protein levels declined rapidly within the first minute. The final detectable band for CW was observed after 20 minutes, at which point only about 2% of the protein remained (**Supplementary Figure S5**; **Supplementary Table S1**). For CM, the last detectable signal appeared after 6 minutes, with less than 1% of the protein remaining (**Figures 5**, **S5**; **Supplementary Table S1**). This highlights the difference in stability between wild-type and mutant parvalbumin (Freidl et al., 2020). Next, an equivalent amount of wild-type and mutant parvalbumin, encapsulated within the core of PBs, was subjected to simulated gastric digestion. As shown in **Figure 5** and **Supplementary Table S1**, it took 90 minutes for core-encapsulated mutant parvalbumin to be fully digested, and core-encapsulated wild-type parvalbumin remained detectable for even longer. Thus, encapsulation in the PB-core prolonged the persistence of both the wild-type and mutant allergen, with average signal intensities of 80% and 40% remaining after 20 minutes of digestion, respectively. Even after 90 minutes, both allergen variants were still detectable (**Supplementary Table S1**). We also compared the persistence of the shell-encapsulated allergen in a simulated digestion process. In contrast to core encapsulation, in which the amount of allergen decreased steadily, the allergens incorporated in the shell exhibited a clear and sudden drop in abundance after about 20 minutes in the gastric phase. Nevertheless, the shell-incorporated wild-type and mutant allergens exhibited greater stability than their soluble counterparts, with an average of 47% and 26% of the signal remaining after 20 minutes, respectively (**Figure 5**, **Supplementary Table S1**).

### 3.3 Uptake of the PBs into Caco-2 cells

**Figure 6:**
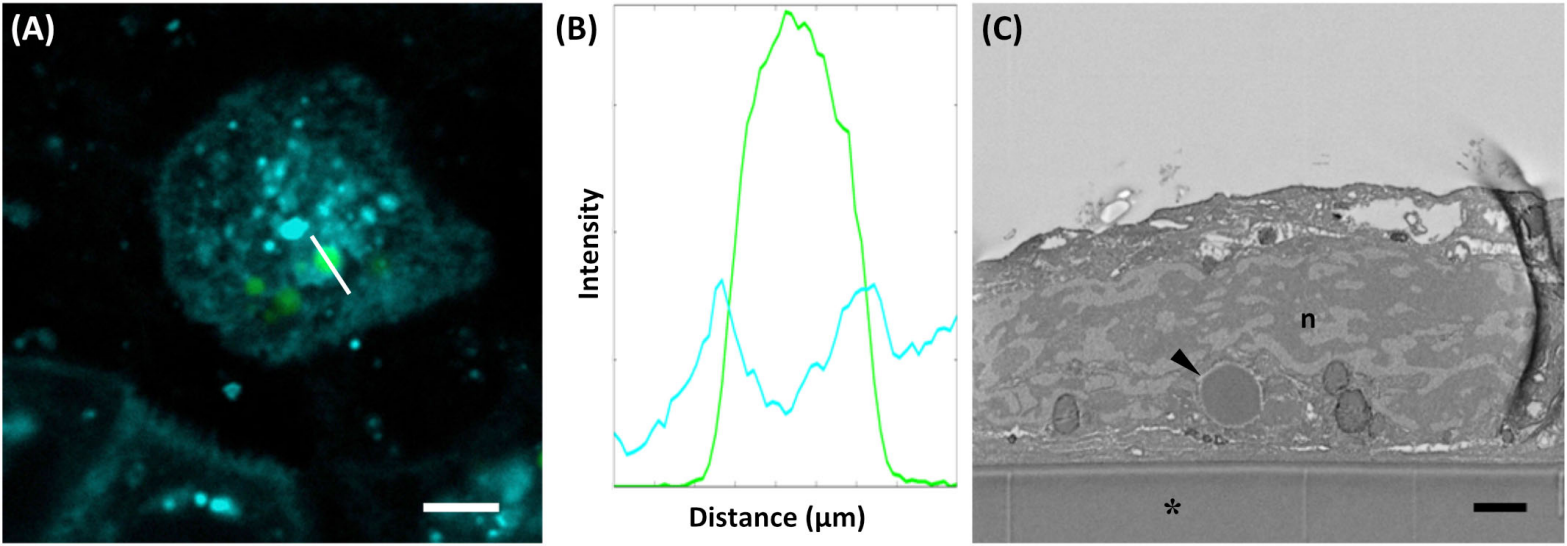
Uptake of PB by Caco-2-cells. **(A)** Cellbrite (cyan), visualizing intracellular membranes, and protein bodies (green). **(B)** Fluorescence intensity profile of the line indicated in **(A)**. Fluorescence intensity corresponds to the readout of the Leica LAS Software. **(C)** SEM cross section of a Caco-2 cell revealing protein bodies within the cytoplasm (arrowhead). Aclar foil (*), nucleus (n). Bars 5 µm **(A)**, 1 µm **(C)**.

To investigate whether multi-layered PBs can be internalized by intestinal epithelial cells, we exposed Caco-2 cells to the different PBs. To analyze their uptake into Caco-2 cells we employed various staining and microscopy techniques. After incubation in a PB suspension (core encapsulation of wild-type parvalbumin), CellBrite^®^ was used to visualize intracellular membranes. A fluorescent membrane surrounding the PB was visible (**Figure 6A**), suggesting PB uptake by endocytosis. Indeed, the fluorescence intensity profile of this region showed a green peak, originating from Zera-EGFP and defining the perimeter of the PB. It was flanked by two additional peaks corresponding to CellBrite^®^ labelled membranes (**Figure 6B**). PB uptake could also be confirmed by electron microscopy. Cross sections of Caco-2 cell monolayer cultures showed PBs enclosed within a membrane and located inside the cells (**Figure 6C**).

## 4 Discussion

This study presents the design of multi-layered PBs in *N. benthamiana* as a novel platform for allergen encapsulation. These structures offer promising advantages for oral immunotherapy, including enhanced control over allergen release and dosage, which may contribute to more effective and individualized allergy management. The successful generation of multi-layered PBs was achieved by the co-expression of three zeins: 16-kDa-γ-zein, Zera, and 15-kDa-β-zein, fused either to fluorescent tags or variants of the major fish allergen parvalbumin. As a result, PBs with distinct core and shell layers were formed, allowing precise allergen localization within the protein matrix. Confocal microscopy confirmed the clear layering of fluorescent labels, demonstrating the structural integrity of the PBs. Additionally, CLEM and immunofluorescence analysis showed the localization of the allergens in defined layers within the PBs. By combining high-resolution imaging, immunogold labelling and immunofluorescence, the exact positioning of the parvalbumins could be mapped, even in the absence of fluorescent labels.

One key finding of this study is the significantly increased resistance of PB-encapsulated parvalbumins to proteolytic degradation compared to their soluble counterparts. This characteristic is a critical factor for inducing robust and sustained oral tolerance (Freidl et al., 2020). We demonstrated that the soluble parvalbumin variants were no longer detectable after twenty minutes for the wild-type parvalbumin and after six minutes for the mutant parvalbumin during simulated gastric digestion. In contrast, core-encapsulated wild-type protein (CWF) remained detectable even after 60 minutes, and this was also observed for the core-encapsulated parvalbumin mutant (CMF), albeit to a lesser extent. Shell encapsulated parvalbumins demonstrated moderate stability, initially resisting proteolysis, but showing a sharp decline in abundance after twenty minutes. Nevertheless, even CMF remained detectable throughout the simulated gastric digestion. This enhanced protection against enzymatic gastric degradation is particularly important for oral administration, as the harsh conditions in the stomach often compromise the efficacy of protein therapeutics including mutant parvalbumin (Freidl et al., 2020; Han et al., 2024). Furthermore, the stability during the intestinal digestion phase was evaluated. While core encapsulation provided prolonged protection also against the simulated intestinal conditions, a gradual decrease in parvalbumin levels was observed over time, indicating the progressive degradation of the protein. The continued persistence in both the gastric and intestinal phases highlights the potential of PBs as a delivery platform for therapeutic proteins requiring prolonged stability. The precise positioning of allergens within specific PB layers provides opportunities to fine-tune the release profile of encapsulated proteins. Thus, core encapsulation may support sustained release, while shell encapsulation might promote faster release.

Beyond stability, effective oral administration requires that the PBs are internalized by human intestinal epithelial cells, a key step for systemic absorption (Mohammed et al., 2022). This was simulated in our study by using Caco-2 cells, an established model for human intestinal epithelial cells (Bailey et al., 1996). Microscopy revealed that the PBs were surrounded by a fluorescently labelled membrane after internalization by Caco-2 cells, indicating uptake by endocytosis. Electron microscopy further confirmed that the PBs were present in intracellular vesicles within the Caco-2 cells. These results are in line with previous research that demonstrated the uptake of the peanut allergen Ara h1 into monocytes using microscopy, showing their internalization into vesicles that are likely part of the endolysosomal pathway (Price et al., 2017). This is a crucial step for antigen presentation and immune activation (van Kasteren and Overkleeft, 2014). While further studies are needed to understand the mechanisms involved in the internalization of PBs by different cell types, the routing of PBs through the endolysosomal pathway supports their suitability for allergen immunotherapy.

Encapsulation of proteins for oral immunotherapy has proven advantageous in other studies using micro- or nanoparticles for this purpose (Jazayeri et al., 2021). Chimeric virus-like particles (VLPs), such as those based on hepatitis E (Jariyapong et al., 2013), Norwalk virus (Ball et al., 1998), HIV env (Takamura et al., 2004) and influenza (Serradell et al., 2019), have demonstrated protease resistance and strong immune responses after oral administration in animal models. Similarly, plant-made enveloped eBioparticles (eBPs) have been studied as an allergen expression platform in recent years. They are capable of inducing IgG responses and modulating dendritic cell activity in mice after administration (Busold et al., 2024; Gomord et al., 2020). In another study, eBPs carrying the peanut allergen Ara h 2 were produced in *N. bethamiana* with *A. tumefaciens*. After incubating sera from Ara h 2-sensitized patients with the eBPs, they measured a significant reduction in IgE binding potency and therefore an extensive decrease in the induction capacity of effector cell-driven allergic responses (Castenmiller et al., 2023). While VLPs have shown remarkable immunogenicity and some protection from gastrointestinal degradation (Serradell et al., 2019), PBs offer distinct advantages. PBs are fully plant-derived and self-assembled without the need for viral components, eliminating some biosafety concerns and simplifying downstream processing (Schwestka et al., 2023). Unlike VLPs, which often require complex multi-protein assembly and precise stoichiometry (Qin et al., 2023), PBs can form from a single fusion construct and are amenable to modular engineering (Hofbauer et al., 2016; Schwestka et al., 2023). Furthermore, PBs based on prolamin storage proteins such as zeins display natural resistance to digestive enzymes and acidic conditions, making them especially suitable for oral delivery (Luo et al., 2023). While VLPs and eBPs are well-established for injectable or mucosal applications, their production is often associated with challenges related to cost and regulatory complexity (Chen and Lai, 2013). PBs are based on cereal storage proteins that are part of the food chain and generally regarded as safe, and they can be rapidly produced in *N. benthamiana* by transient expression. Additionally, their multi-layered architecture offers new opportunities for tailoring antigen localization and release profiles.

PBs provide a particularly versatile platform for the *in planta* encapsulation of allergens. For example, endogenous PBs were utilized in a study where a hypoallergenic Bet v 1 tolerogen against birch pollen allergy was specifically expressed in the endosperm tissue of transgenic rice seeds (Wang et al., 2013). Fukuda et al., 2018 incorporated hypoallergenic forms of the major Japanese cedar pollen allergens into transgenic rice seed PBs. Oral immunotherapy in mice performed with such PBs led to the suppression of pollen-induced allergic conjunctivitis. Furthermore, these PBs even showed a protective effect against the development of allergic conjunctivitis, in experiments, where the transgenic rice was fed to mice prophylactically (Fukuda et al., 2018). Although the use of stable transgenic plants, particularly edible species, presents some advantages, their development remains a time-consuming and costly process. Moreover, the presence of endogenous storage proteins complicates the design of well-defined, multi-layered PBs. In contrast, transiently expressed PBs can be produced more rapidly and under controlled conditions, enabling the incorporation of the protein of interest either within the PB core or exposed on its surface. In addition, transient expression systems based on agroinfiltration are typically capable of achieving higher protein yields, rendering them well-suited for commercial production (Feng et al., 2022; Mardanova and Ravin, 2021; Nosaki et al., 2021; Suzaki et al., 2019).

Our study not only provides an advanced analysis of transiently expressed PBs for the encapsulation of allergens but also highlights several remaining issues. While our current findings are encouraging, they are based on *in vitro* analyses. Thus, *in vivo* studies using animal models are required to assess the efficacy, safety, and immunogenicity of PB-based delivery systems under physiological conditions. To further enhance their performance, the composition of PBs could also be optimized regarding stability and functionality. For example, incorporating alternative zein variants or fusion partners with suitable properties might additionally improve structural integrity under specific conditions. Furthermore, the development of stimuli-responsive PBs could also represent a future improvement towards producing adaptable PBs for diverse therapeutic applications. PB degradation could be triggered for example by certain environmental stimuli such as pH, temperature or enzymatic activity, potentially promoting allergen release under specific environmental conditions. However, for such advanced applications involving targeted delivery, a deeper understanding of the cellular mechanisms governing uptake, transport and degradation of PBs will be essential.

In conclusion, we have shown in this study that multi-layer PBs represent a robust and versatile system for the encapsulation of allergens. We were able to demonstrate precise localization of the major fish allergen derivatives, their enhanced stability during simulated gastric and intestinal digestion, and successful endocytic uptake. These findings highlight the potential of PBs as a delivery platform and provide a foundation for the further development of PB-based systems. Future animal studies using murine allergy models will be an essential next step to further evaluate the relevance of the platform for therapeutic strategies targeting allergic and other immune-related diseases.

## Data Availability

The original contributions presented in the study are included in the article/**Supplementary Material**. Further inquiries can be directed to the corresponding authors.
