# Supplementary material for: Bio-encapsulation of allergen-derivatives for specific immunotherapy
Source: Front Plant Sci. 2025 Sep 2;16:1652246. doi: 10.3389/fpls.2025.1652246 (PMC12436285; doi:10.3389/fpls.2025.1652246)
Supplement: Supplementary file 1 [file DataSheet1.pdf]

Supplementary Material

Bio-encapsulation of allergen-derivatives for specific immunotherapy

Fabian Schubert[1*], Elsa Arcalis[1], Maximilian Kyral[1], Barbara Jeitler[2], Marianne Raith[2], Ines Swoboda[2], Eva Stoger[1*]

*** Correspondence:** fabian.schubert@boku.ac.at; eva.stoeger@boku.ac.at

1 Supplementary Figures

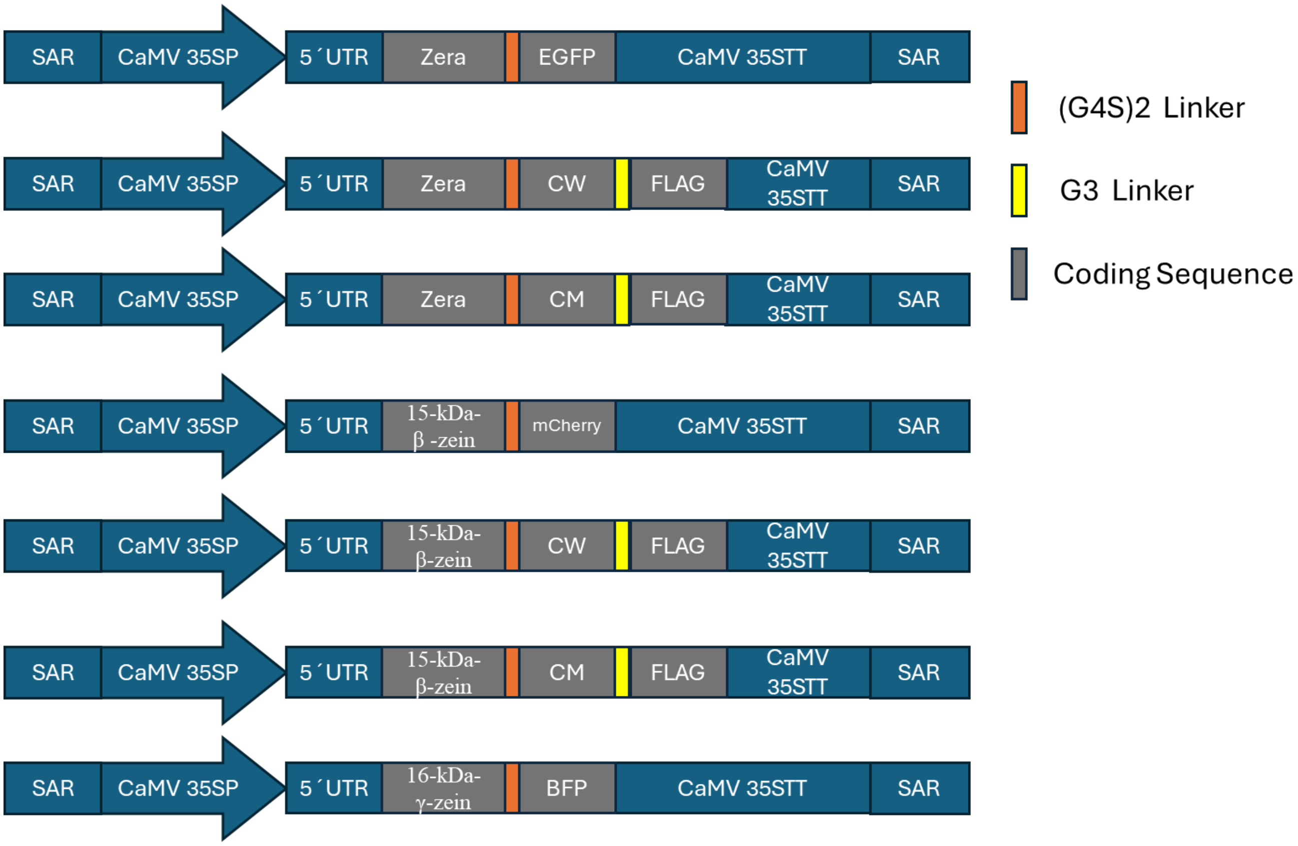

Supplementary Figure S1. Zein constructs. All zein constructs contain their native signal peptide. Each coding sequence features a $(GGGGS)_2$ linker, shown in orange, connecting the zein to either the fluorophore or the allergen wild-type and mutant protein. For constructs with a C-terminal flag-tag, the tag is attached via a G_3 linker (shown in yellow). SAR: Scaffold attachment region, CaMV 35SP/TT: promoter/terminator with duplicated enhancer and terminator of the Cauliflower mosaic

virus (CaMV) 35S gene; 5'UTR: untranslated region of the tobacco etch virus. CW= Cyp c 1 wildtype, CM= Cyp c 1 mutant, Zera= N-terminal part of 27-kDa-γ-zein.

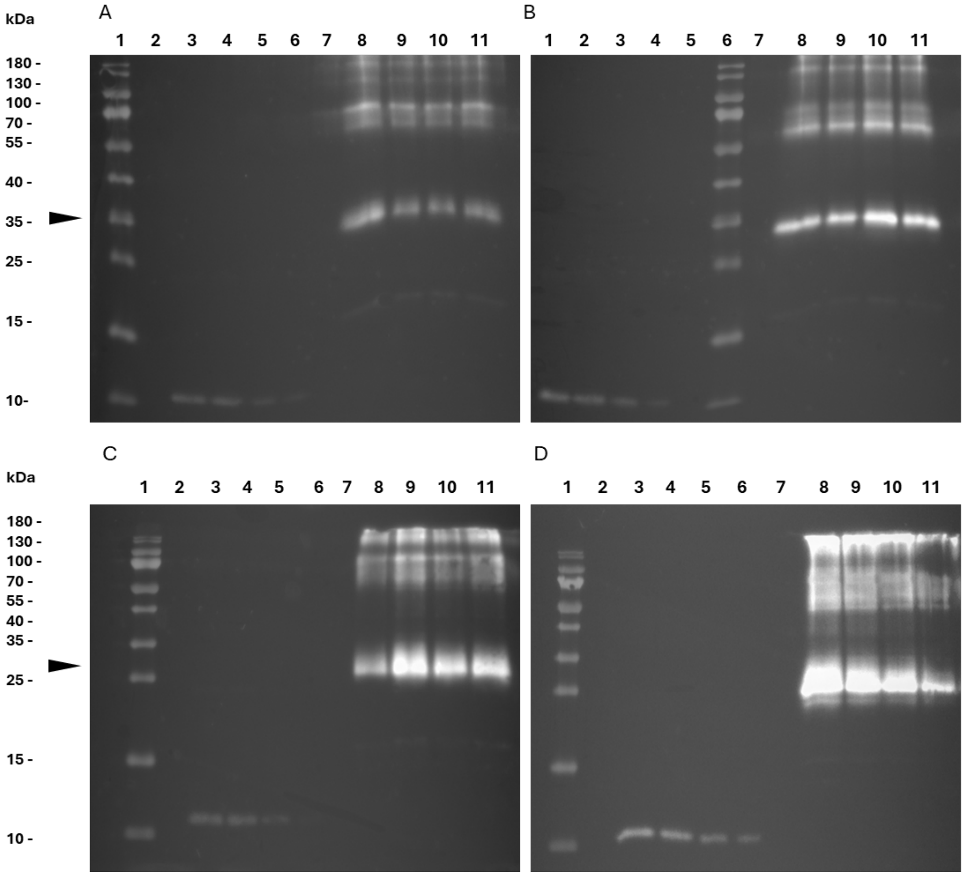

Supplementary Figure S2. Immunoblot detection of parvalbumin derivatives in isolated multilayered protein bodies. A) Core-encapsulation of the15-kDa-β-zein-CWF fusion protein. Lane 8-11 isolated protein bodies (16.8mg leaf tissue). Lane 3-6, positive control (62.5ng, 50ng, 25ng and 12,5ng of soluble wild-type parvalbumin at 12 kDa). B) Core-encapsulation of the15-kDa-β-zein-CMF fusion protein. Lane 8-11 isolated protein bodies (16.8mg leaf tissue). Lane 1-4, positive control (62.5ng, 50ng, 25ng and 12,5ng of the soluble parvalbumin mutant protein). C) Shell-encapsulation of the Zera-CWF fusion protein. Lane 8-11 isolated protein bodies (16.8mg leaf tissue). Lane 3-6, positive control (62.5ng, 50ng, 25ng and 12,5ng of soluble wild-type parvalbumin). D) Shell-encapsulation of the Zera-CMF fusion protein. Lane 8-11 isolated protein bodies (4mg leaf tissue). Lane 3-6, positive control (62.5ng, 50ng, 25ng and 12,5ng of soluble parvalbumin mutant). Primary antibody: antiserum raised against the carp parvalbumin mutant, recognizing both, CM and CW. Secondary antibody: anti-rabbit-HRP. Chemiluminescence detection. The arrows indicate the expected molecular weight of the fusion protein A) 15-kDa-β- zein-CWF, B) kDa-β- zein-CMF, C) Zera-CWF D) Zera-CMF.

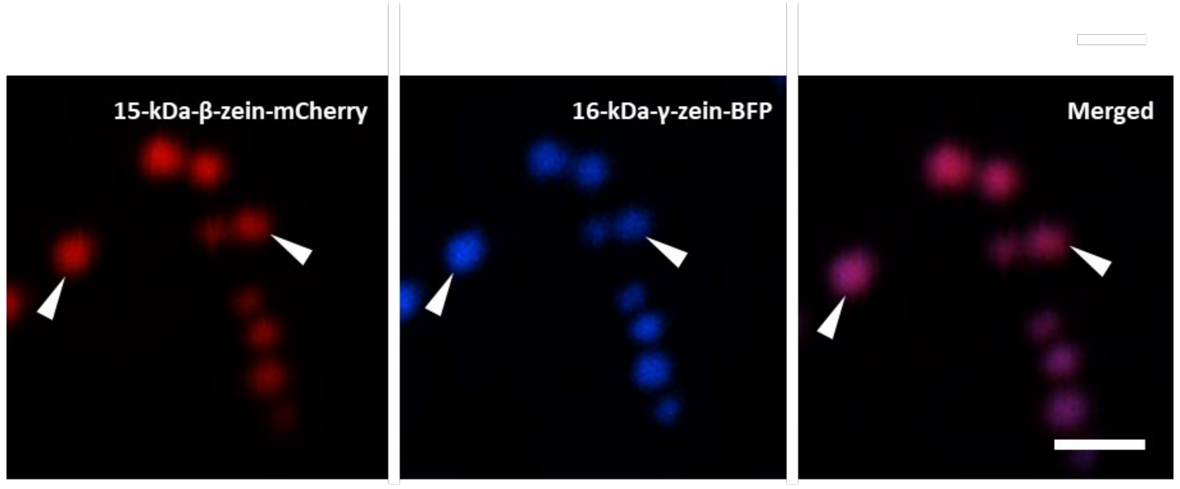

Supplementary Figure S3. Shell encapsulation of mutant parvalbumin. Co-expression of Zera-CMF, 16-kDa-γ-zein-BFP, and 15-kDa-β-zein-mCherry. CLSM, 15-kDa-β-zein and 16-kDa-γ-zein-BFP form the core of the protein bodies. Bar 1 μm

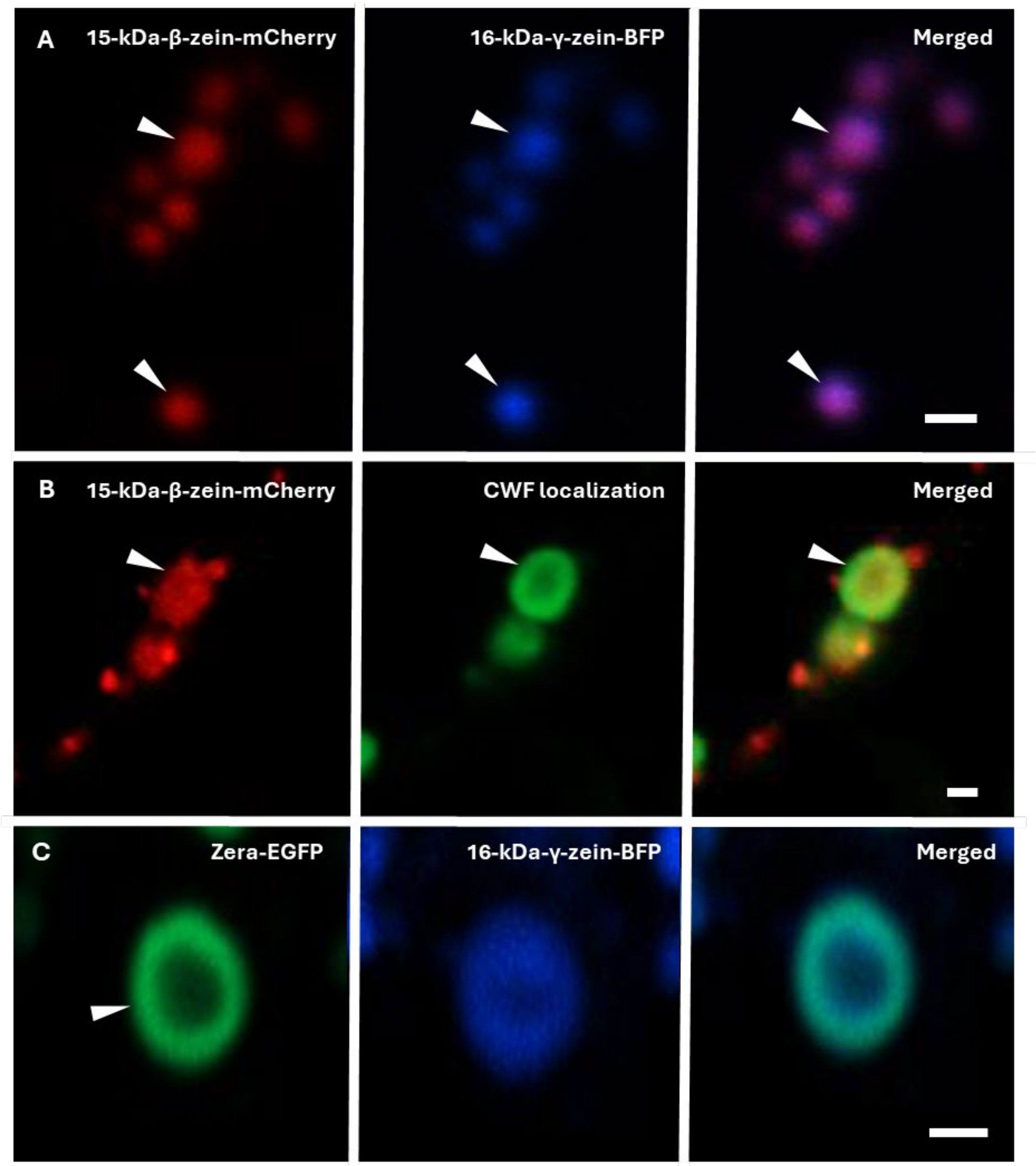

Supplementary Figure S4. Shell encapsulation of wild-type parvalbumin. Co-expression of Zera-CWF, 16-kDa-γ-zein-BFP, and 15-kDa-β-zein-mCherry. A) CLSM images; first panel:15-kDa-β-zein-mcherry; second panel: 16-kDa-γ-zein-BFP; third panel: overlay images. B) Immunolocalization images; first panel:15-kDa-β-zein-mcherry; second panel: detection of wild-type parvalbumin with rabbit anti- parvalbumin antibody visualized with donkey anti-rabbit antibody conjugated to Alexa Fluor 488 (shown in green); third panel: overlay images. C) CLSM images; first panel: Zera-EGF; second panel: 16-kDa-γ-zein-BFP; third panel: overlay images. Bars 1 μm.

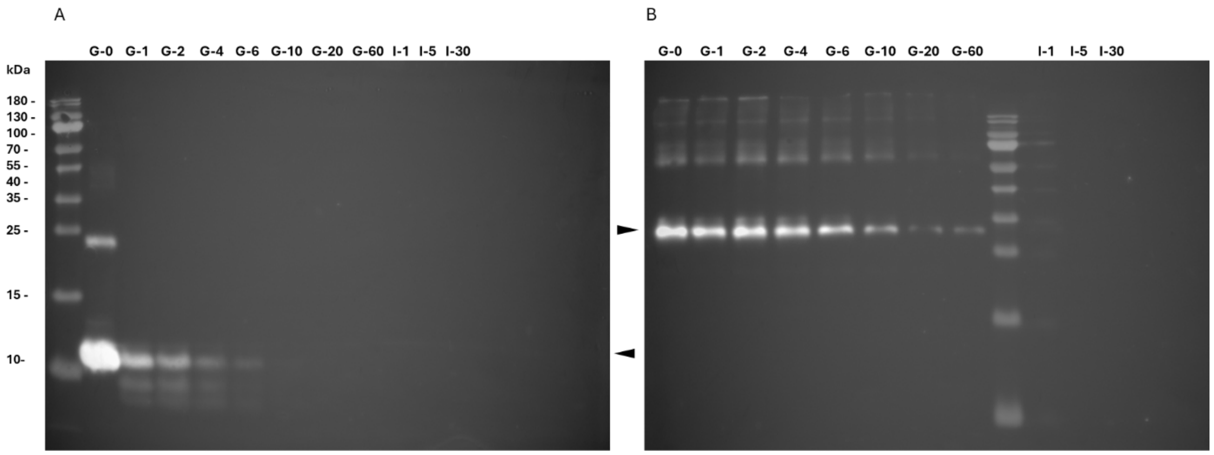

Supplementary Figure S5. Representative example for immunoblot detection of parvalbumin after simulated gastric digestion. A) digestion of soluble parvalbumin mutant protein (CM), B) digestion of Zera-EGFP + 16-kDa-γ-zein-BFP + 15-kDa-β-zein-CM protein bodies (core encapsulated parvalbumin mutant). G-0 through G-60 represent the gastric phase of simulated digestion, whereas I-1 through I-30 represent the intestinal phase (number corresponds to minutes of digestion). Each lane was loaded with aliquots of the reaction corresponding to 20 mg of plant tissue/5 µg of protein at the start of the digest. Primary antibody: Rabbit antiserum raised against the carp parvalbumin mutant. Secondary antibody: anti-rabbit-HRP. Detection using chemiluminescence. The arrows indicate the expected molecular weight of the soluble CM (A) and the fusion protein kDa-β- zein-CMF (B).

Supplementary Table 1: The average percentage of parvalbumin allergen remaining after defined periods of *in vitro* digestion were calculated for each encapsulation variant.
n.d.: non detectable; WT: wild-type parvalbumin; MT: mutant parvalbumin.

	Minutes of *in vitro* digestion			
	0	20	60	90
**Core encapsulation WT**	100%	80%	61%	20%
**Shell encapsulation WT**	100%	47%	19%	12%
**Core encapsulation MT**	100%	40%	29%	5%
**Shell encapsulation MT**	100%	26%	5%	n.d.
**Soluble WT allergen**	100%	2%	n.d.	n.d.
**Soluble MT allergen**	100%	n.d.	n.d.	n.d.
